# Severe Esophageal Stricture Post Accidental Corrosive Substance Ingestion: A Case Report of Balloon Endoscopic Dilation

**DOI:** 10.1155/2022/8520213

**Published:** 2022-07-06

**Authors:** Leen Jamel Doya, Maria Naamah, Hanin Ahmed Mansour, Ali Ibrahim, Ammar Omran

**Affiliations:** ^1^Department of Pediatrics, Tishreen University Hospital, Lattakia, Syria; ^2^Department of Pediatrics, Gastroenterology and Hepatology, Tishreen University Hospital, Lattakia, Syria; ^3^Pediatric Surgery, Department of Pediatric Surgery, Tishreen University Hospital, Faculty of Medicine, Lattakia, Syria

## Abstract

Corrosive substance ingestion is a very serious home accident, mostly common in developing countries. It frequently causes esophageal burns in the acute stage and esophageal stricture, stenosis, and even cancer in the chronic stage. Severe cases of caustic esophagitis may require esophageal replacement. We describe a case of balloon endoscopy dilation in a two-year-old girl with a severe stricture of the esophagus resulting from accidental ingestion of a corrosive substance (strong alkaline liquid) which helped the patient preserve the esophagus and prevent esophageal replacement. We describe the clinical complication and development during the treatment.

## 1. Introduction

Caustic ingestion (CI) in children is a serious medical emergency that leads to serious complications with a peak incidence at 2 years of age [1]. It is considered as one of the main causes of death in children less than 5 years. The prevalence of CI in our area is unknown. However, 4.8% of the medical admissions annually were associated with CI in children [2]. CI includes either acids or alkali that causes injuries that vary from minimal to severe. Alkaline substance ingestion (ASI) such as NaOH, drain openers, and bleaches are the most common cases of CI in western countries, while acid substances are more common in countries like Syria [3]. ASI is more tasteless and odorless than acidic products resulting in ingestion of larger quantities and increased risk of serious injury. Acid products cause coagulation necrosis with coagulum layer formation that limits the depth of injury. They also cause less esophageal injury and more gastric injury [4]. Corrosive ASI causes liquefaction necrosis with deeper injuries, dehydration of tissues, saponification of fats, and thrombosis of blood vessels [5]. Herein we report a special case of balloon endoscopy dilation in a two-year-old girl with a stricture of the esophagus resulting from accidental ingestion of a corrosive substance which saved the patient from an esophageal replacement surgery.

## 2. Case Report

**Figure 1:**
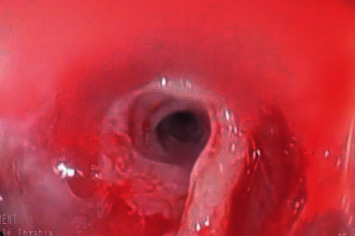
The EGD revealed diffuse erosions with a whitish plaque and hemorrhagic spots along the esophagus.

**Figure 2:**
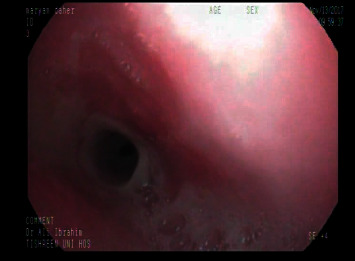
An endoscopy showed worsening stenosis.

**Figure 3:**
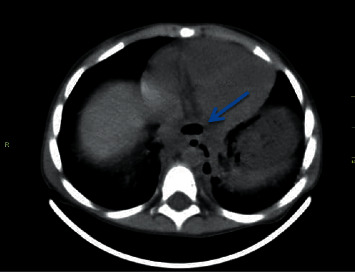
A CT scan showed the esophagus and air bubbles which can be from ruptured esophagus. The esophagus is dilated with thickened walls. It is difficult to see loculated pleural effusion and pulmonary abscess.

A 2-year-old female child was admitted with accidental ingestion of a strong alkaline solution (pH > 9, NaHCO3 96% and 4% KHCO3) and vomiting after taking the substance. Soon after ingestion, she was noted with swelling of lips and tongue followed by drooling and acute onset of epigastric pain. Upon examination, there were erosions of the lower lip, the dorsum of the tongue, and the floor of the mouth with mild tenderness at the epigastric region. The vital signs and the systemic examination were normal. The next day, esophagogastroduodenoscopy (EGD) was performed while the patient was under intravenous anesthesia (midazolam 0.2 mg/kg). The EGD revealed diffuse erosions with a whitish plaque and hemorrhagic spots along the esophagus (grade IIa in the upper third of the esophagus and IIb in the lower third) (Figure 1). The stomach had petechiae at the fundus and the whole body. The patient was given conservative treatment with intravenous fluids and antibiotics. Initially, the child could swallow liquid substances well. However, she developed dysphagia 2 weeks later. EGD revealed two separated strictures at 15 cm and at 18 cm from the edge of the teeth. Endoscopic dilation with a balloon had been done, and the child was discharged with a recommendation of semi-solid feeding with review after 3 weeks for periodic balloon endoscopic dilation if no symptom developed. A repeated endoscopic dilation with the balloon was performed every 14 days, but the stenosis was getting worse (Figure 2). After one of the dilation sessions, the child developed subcutaneous emphysema in the neck. The girl was put on nothing by mouth (NPO), broad-spectrum antibiotics, and intravenous feeding with close monitoring for 14 days. CT scan was performed which showed a posterior mediastinal abscess with an expansion of the thoracic esophageal and fistula behind the esophagus. An incision and drainage operation was performed to treat an abscess. Consequently, the child was discharged and the dilation sessions were continued. 2 months later, the girl was reviewed with a complaint of fever started two weeks ago which was treated with antibiotics without improvement. Her father mentioned during history-taking a story of food impaction that happened one day before the fever started, leading to dyspnea, during which the father performed the Heimlich maneuver successfully. A CT scan was performed which showed the esophagus and air bubbles which can be from ruptured esophagus. There was an expansion of the thoracic esophagus with thickened walls. No loculated pleural effusion or pulmonary abscess in the medial section of the left lower lobe was observed (Figure 3).

**Figure 4:**
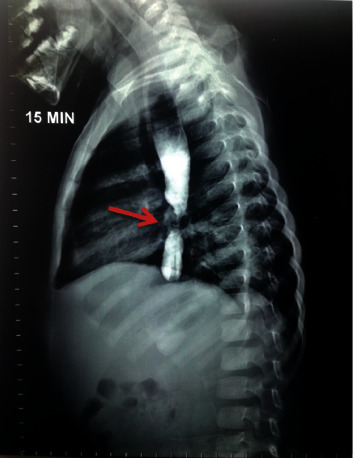
Barium esophagogram showed esophageal dilation with severe narrowing in the lower esophagus (red arrow).

**Figure 5:**
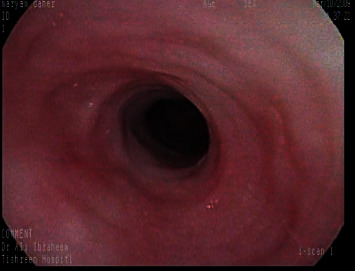
An esophagogastroduodenoscopy showed normal esophageal findings.

Broad-spectrum antibiotics were given with intravenous feeding, and gastrostomy was performed. The girl was discharged after 12 days with feed via gastrostomy. Barium esophagography was done which showed esophagus dilation with severe narrowing in the lower esophagus (Figure 4). A repeated endoscopic dilation with the balloon was performed after this acute episode every 14 days, then every month, and every two months, over 2.5 years. The gastrostomy tube was removed after normalization of the esophagus with a return to oral nutrition. Currently, the girl is normal with no dysphagia (Figure 5). Annual follow-up is done clinically and endoscopically.

## 3. Discussion

Clinical manifestation of ASI may vary from no symptoms to nausea, vomiting, abdominal pain, dysphagia, odynophagia, chest pain, or stridor. Even if the examination is normal or the patient is asymptomatic, it may cause severe esophageal burns as a short-term effect, and esophageal stricture, perforation, obstruction, and cancer as long-term effects [1]. Gastroenterology tract mucosa after CI could be most efficiently assessed by EGD which should be performed during 12–24 hours of ingestion [6]. Endoscopically, esophageal injury after CI is graded with a score of grade 0 for no injury, grade I for mucosal edema and hyperemia, grade IIA for superficial ulcers and bleeding, grade IIB for deep focal or circumferential ulcers, and grade IIIb for significant circumferential injury with ulcers and extensive necrosis [7]. Since 1981 when London et al. reported successful balloon catheter dilation (BCD) treatment for esophageal strictures, it has become the worldwide preferred treatment for severe gastrointestinal strictures [8]. The overall success rates of BCD have been reported to range from 67 to 98%, whereas the rupture rates have been reported to range between 0 and 9% [7].

BCD is the safest, most effective, and less traumatic procedure although some patients may develop recurrent strictures after dilation and others have refractory strictures requiring multiple dilations. On the other hand, endoscopic dilations may cause a significant risk of perforation (15–20%) and the development of new strictures [9].

If BCD fails, surgery in the form of esophageal replacement using stomach or colonic interposition should be considered. Mortality and morbidity following surgery are low in expert hands [9].

Our present case report describes a patient who developed severe corrosive esophagitis after ingesting a strong alkaline solution that developed into esophageal strictures. Initially, BCD was believed to be the treatment of the choice. Although the patient was severely constricted and exposed to severe complications, the condition was managed with repeated dilatation over several months and the patient did not need esophageal replacement.

## Data Availability

All data generated or analyzed during this study are included within this article.
